# Combined neonicotinoid pesticide and parasite stress alter honeybee queens’ physiology and survival

**DOI:** 10.1038/srep31430

**Published:** 2016-08-31

**Authors:** Claudia Dussaubat, Alban Maisonnasse, Didier Crauser, Sylvie Tchamitchian, Marc Bonnet, Marianne Cousin, André Kretzschmar, Jean-Luc Brunet, Yves Le Conte

**Affiliations:** 1 INRA, UR 406 Abeilles et environnement, 84914, Avignon, France; 2 INRA, UR 546 Biostatistique et Processus Spatiaux, 84914, Avignon, France; 3 UMT PrADE, Site Agroparc, CS 40509, Avignon, France; 4 ADAPI (Association pour le développement de l’Apiculture), 22, Avenue Henri Pontier, 13326, Aix en Provence Cedex 1, France

## Abstract

Honeybee colony survival strongly relies on the queen to overcome worker losses exposed to combined stressors like pesticides and parasites. Queen’s capacity to withstand these stressors is however very little known. The effects of the common neonicotinoid pesticide imidacloprid in a chronic and sublethal exposure together with the wide distributed parasite *Nosema ceranae* have therefore been investigated on queen’s physiology and survivorship in laboratory and field conditions. Early physiological changes were observed on queens, particularly the increase of enzyme activities (catalase [CAT] and glutathione-S-transferase [GST] in the heads) related to protective responses to xenobiotics and oxidative stress against pesticide and parasite alone or combined. Stressors also alter the activity of two other enzymes (carboxylesterase *alpha* [CaE *α*] and carboxylesterase *para* [CaE *p*] in the midguts) involved in metabolic and detoxification functions. Furthermore, single and combined effects of pesticide and parasite decrease survivorship of queens introduced into mating hives for three months. Because colony demographic regulation relies on queen’s fertility, the compromise of its physiology and life can seriously menace colony survival under pressure of combined stressors.

Survival of honeybee colonies strongly relies upon the queen, the one individual whose life is crucial for colony development^1^. In the nature, the queen and the whole colony are constantly under the pressure of multiple stressors like agro-chemicals, pathogens and poor food resources, suspected to underlie the decline of their populations around the world^2^. Compared with workers, queens usually live longer, which means they can be exposed to environmental stressors over long time periods with lethal consequences. The loss of the queen seems to be the Achilles heel of superorganism resilience, the ability to tolerate the loss of somatic cells (worker bees) as long as the germ line (reproduction) is maintained. It therefore represents a key factor to understanding colony fate challenged by combined environmental factors^3^. Resilience was recently demonstrated in field conditions where individual honeybees near neonicotinoid-treated fields disappeared at a faster rate. The impact of this was however buffered by the colonies’ demographic regulation response^4^.

Insecticides from the neonicotinoid class have been in the center of controversy for the last years due to their high toxicity to non-target organisms like domestic and wild pollinators^2^. Because of their systemic action, they can reach bee food resources like pollen and nectar of treated crops^2^,^4^,^5^. The negative effects of neonicotinoids have been demonstrated, including imidacloprid on insect pollinators^5^,^6^,^7^,^8^, even though honeybee colony field trials have been incapable of detecting laboratory predicted sublethal effects with conventionally accepted levels of certainty^9^. The interactive effects between neonicotinoids and pathogens are now also well known in honeybees and bumble bees^2^. However, in natural conditions interaction effects are not clearly demonstrated^10^. Particularly, in honeybees, the impact of the endoparasite *N. ceranae* and imidacloprid were proved to be enhanced when both stressors were present^11^. Interactions between imidacloprid and *N. ceranae* can be expected, as both have the potential to disturb similar metabolic functions related to immunity, energetic resources and antioxidant responses^11^,^12^,^13^.

In honeybee queens, a few recent studies show increased supersedure (the natural process by which an old or failing queen is replaced), probably driven by a reduced reproductive performance in neonicotinoid exposed honeybee queens^6^,^14^. In bumble bee colonies, similar exposure decreased queen’s production and survival by 85% and 37% respectively associated to reduced workers foraging efficiency^15^,^16^. In bumble bee queens, a significant interaction that increased mortality was also observed between the parasite *Crithidia bombi* and a neonicotinoid^17^. Given this, we hypothesized that honeybee queens chronically exposed to an environmental relevant sublethal dose of imidacloprid during their first days of life, in combination with the common parasite *N. ceranae*, experience physiological changes and shorter lives. To test this hypothesis, survivorship trials were carried out: four groups of queens were settled in the laboratory: *N. ceranae* infected queens (N), imidacloprid exposed queens (I), both infected and pesticide exposed queens (NI) and non-treated queens as controls (C). The same experiment was repeated in 2010 and 2012. Queens from groups N and NI only were inoculated orally at birth with 200,000 spores/queen of *N. ceranae,* as infection process starts in the midgut after spore ingestion^13^. Queens were then kept in cages with a group of attendance bees^18^. Same spore charge under similar conditions was proved to be sublethal to queens in cage experiments during comparable time lapse^18^. Queens from groups I and NI were then exposed for 8 days to sugar syrup contaminated with 0.7 μg/l of imidacloprid^11^. Similar levels of imidacloprid were found in royal jelly (0.6 μg/kg in average) produced by colonies fed with contaminated diet patties after a chronic sublethal field exposure mimicking imidacloprid treatments applied to crops during bloom^5^. Because in nature a queen rarely feeds herself, but is fed by the attendance bees (nurses) with the royal jelly they produced in their hypopharyngeal glands, we expected an indirect exposure of queens to the pesticide^1^. After exposure, queens were transferred into small hives used to rear queens (mating nuclei) placed in the field to naturally mate and lay eggs for 3 months (Fig. 1).

## Results and Discussion

Survivorship curves (Fig. 2A,B) show the negative effect of combined stressors, while the impact of each stressor alone varied between the two years. On 2010, I, N and NI had statistically similar survivorship between them, but were all significantly lower than C. On 2012, C, I and N were statistically similar, and only NI was significantly different from C. That year, 66.7% of the control queens were accepted after introduction into the nuclei (Fig. 2B), which is considered as a common yield in beekeeping^19^. This control mortality was kept unchanged in the statistical analysis (see Methods). Survivorship curves (Fig. 2A,B) allow also inferring the queens’ median lifespan (T50). In 2010 and 2012, 50% of the queens from NI group were dead 45 and 15 days after introduction in the nuclei respectively. These were the lowest T50 from both years with exception of N on 2012 which also showed a T50 of 15 days. Queens from I group had a T50 of 75 days both years as well as N group from 2010. Survivorship differences between years might be related to the genetic background of sister queens used in the trials, that each year were reared from a different source colony accounting for genetic variability. In fact, the susceptibility to neonicotinoids can vary depending on the genetic basis of queens^6^. The variable susceptibility of honeybees of different genotypes to neonicotinoids has also been observed among worker bees^20^. Particularly imidacloprid sensitivity can change from one honeybee colony to another because of differences in the oxidative metabolism^8^. Regarding *N. ceranae*, our results show that early infections in queens can seriously compromise survival, which agrees with Chaimanee *et al*.^21^ who observed that young queens are more susceptible to the parasite than older queens because the immune responses to *N. ceranae* develop as queens age. Supersedure of queens is suspected to be due to changes induced by *N. ceranae* on the queen mandibular pheromones (QMP) that regulates the functioning of the colony stimulating queen attendance by workers, inhibiting worker ovary development and regulating worker behavioral maturation^18^. The observed differences between years might also be linked to environmental factors like climate that can impact the quality of honeybee food resources which can negatively affect bee’s immunity^2^.

To address physiological responses to stressor agents, infected or non-infected 8-days-old queens not yet mated, were analyzed just after pesticide exposure to a set of biochemical markers already tested on worker bees: CAT with a strong antioxidant action, GST involved in multiple metabolic functions including antioxidant activity and CaE *α* and CaE *p* implicated in detoxification^22^,^23^. GST in head tissue is clearly induced by imidacloprid exposure and not by *N. ceranae* infection (Fig. 3A) despite GSTs have antioxidant functions and that parasite infection induces oxidative stress^13^, (ANOVA, N = 43, F = 18.83, p – value = 1.04e-07; Tukey post-hoc test at 95% confidence level, C vs. I: p – value = 0.0000255; C vs. N: p – value = 0.7537960; C vs. NI: p – value = 0.0000026; N vs. I: p – value = 0.0003927; N vs. NI: p – value = 0.0000352; I vs. NI: p – value = 0.7522841). The observed activity in imidacloprid exposed queens is probably related to phase-II GST enzyme involved in detoxification process, which transforms xenobiotics in more polar compounds to be excreted or further metabolized^24^,^25^. In fact, GSTs don’t participate directly in the detoxification of imidacloprid which is mainly metabolized by phase-I enzymes^25^. Nevertheless other metabolic functions might be also affected because the superfamily of GST enzymes is composed of six different types, some of them found to be well represented in honeybee brain tissue^24^. Unfortunately, its specific function there remains unknown^24^. In contrast GST activity in the midgut of treated queens is very variable and no significant differences were observed between groups, though there is a general downward trend that seems to be induced by each stressor especially *N. ceranae* (Fig. 3B) (ANOVA, N = 43, F = 2.244, p – value = 0.0984). Concerning the pesticide, the lack of significant differences of GST activity in the midgut of treated queens compare to C was not expected considering that in honeybee species GST is mainly localized in that organ which is the main place of metabolisation of imidacloprid^25^. One explanation can be that two of the more toxic imidacloprid metabolites responsible for lethal effects, olefin derivative and 4/5- hydroxyimidacloprid, are detected in the head rather than the midgut, in addition to 3 other metabolites, all of them with a long persistence^25^. In the midgut, only 3 less toxic compounds are present^25^. However, to our knowledge, the metabolism of imidacloprid has been studied only in worker bees^25^ and differences in the physiology between workers and queens can underlie different responses. In fact, Dahlgren *et al*.^26^ observed that for some acaricides queens are more tolerant than workers. Apart from imidacloprid, workers also respond differently than queens to *N. ceranae* infection by increasing GST activity in the midgut to fight oxidative stress^13^, contrary to the decreasing tendency we observed in queens (Fig. 3B). This result agrees with observations that queens and workers might have different responses against infection. For example, workers bees infected by *N. ceranae*, show lower levels of gene expression of vitellogenin (Vg), that probably render bees more susceptible to infection, as Vg is a yolk protein capable to reduce oxidative stress^12^. In turn, in infected queens, Vg titers and antioxidant capacity increase, which might help to fight infection^18^. Although our results represent the physiological response at a specific point in time, early in the life of queens, one could expect that the observed tendency to decrease GST activity in the midgut will not recover given the reduction of survivorship of *Nosema* infected queens (Fig. 2A).

Queens under the pressure of both stressors (NI) present a significantly higher CAT activity in the heads, while CAT activity of I and specially N was higher than C but not statistically different (Fig. 3C) (ANOVA, N = 43, F = 3.461, p – value = 0.0253; Tukey post-hoc test at 95% confidence level, C vs. I: p – value = 0.8856685; C vs. N: p – value = 0.2361979; C vs. NI: p – value = 0.0234735; N vs. I: p – value = 0.6440953; N vs. NI: p – value = 0.6081494; I vs. NI: p – value = 0.1139478). Even if infected queens enhance CAT activity (our results) and develop a higher total antioxidant enzyme activity and an elevated Vg titer^18^, it seems these protective responses are not enough to cope with the physiological stress of *N. ceranae* over the long-term. This is especially true when combined with neonicotinoid exposure (Fig. 3C), and is consistent with previous studies^27^. CAT is the primary defense against the overproduction of reactive oxygen species (ROS) that can be originated following *N. ceranae* infection as a defense response. ROS are however not specific and when not sufficiently reduced, they damage the organism by reacting with macromolecules of biological importance such as lipids, proteins, nucleic acids, and carbohydrates, eventually leading to cell death^13^.

CaE *α* activity in the midgut also reflects the stress level of queens, which is significantly more elevated in I and NI compared to C (Fig. 3D), (ANOVA, N = 43, F = 10.52, p – value = 3.33e-05; Tukey post-hoc test at 95% confidence level, C vs. I: p – value = 0.0000167; C vs. N: p – value = 0.1138786; C vs. NI: p – value = 0.0077867; N vs. I: p – value = 0.0114687; N vs. NI: p – value = 0.5681849; I vs. NI: p – value = 0.3053523). CaE *α* is a phase-I enzyme that would therefore be directly involved in the detoxification of imidacloprid or might also responded to the production of other xenobiotics during intoxication and infection processes^22^,^23^,^25^. Additionally, the expression of CaEs during development might also be regulated by other factors such as hormones^28^. However, even in mammalians, the physiological mechanisms which control them are relatively little known^28^. Finally, CaE *p* in the midgut (Fig. 3E) presents a decreasing trend, similar to GST response in the midgut, especially with *N. ceranae* infection (ANOVA, N = 43, F = 4.238, p – value = 0.011; Tukey post-hoc test at 95% confidence level, C vs. I: p – value = 0.620222; C vs. N: p – value = 0.0062021; C vs. NI: p – value = 0.3849647; N vs. I: p – value = 0.1255768; N vs. NI: p – value = 0.3510227; I vs. NI: p – value = 0.9680898). The reduction of CaE *p* activity deserves additional attention since it might render queens more vulnerable to xenobiotics or affect other metabolic functions.

In summary, we show here that both chronic exposure to very low doses of neonicotinoid and infection by a common honeybee parasite, affects queens’ physiology with a trade-off on queens’ survival. Specifically, we found a protective response against imidacloprid through the increased activity of GST in the head and CaE *α* in the midgut. A protective response to oxidative stress was also observed from CAT activity in the head, especially in the case of *N. ceranae* infection combined with imidacloprid. However, in the midgut, which is the first barrier to parasite development and oral pesticide exposure^25^,^27^, there was a decreasing trend in the activity of GST and CaE *p* particularly during infection. This decrease might negatively affect other metabolic and detoxification functions which are probably linked to increased mortality. The reduced lifespan of stressed queens, suggests that the physiological protective responses to oxidative stress and xenobiotics detoxification, observed before mating, are not sufficient in the long term to cope the negative effects of pesticide exposure and parasite infection. This could explain previous reports of queen supersedure^6^, of decreased worker production by the queen^15^, of queen and workers death^27^, as well as the loss of colony resilience^3^, essential to colony survival under adverse conditions^4^.

## Methods

### Experimental design

Four groups of 10 emerging queens from local breeds of *A. mellifera*, were treated as follow: (i) inoculated with spores of *N. ceranae* at emergence (N), (ii) exposed to a sublethal dose of pesticide from emergence until they were 8-days-old (I), (iii) both inoculated with *N. ceranae* spores and exposed to the pesticide (NI), and (iv) neither inoculated with spores nor exposed to the pesticide corresponding to the control group (C). This part of the experiment was performed in laboratory conditions. When the queens were 8-days-old, they were transferred to small hives specially designed to rear queens (mating nuclei) and settled in a non-cultivated field surrounded by an urban forest located at the south-east border of the city of Avignon, in the South of France. The experiment was repeated in 2010 and 2012, between spring and autumn and queens’ survival in the nuclei was monitored for 3 months. Just before transfer to the nuclei, some of the queens were sacrificed for analysis of a set of five physiological biomarkers (see further). Three additional measurements were made: (i) the success of *N. ceranae* experimental infection (n = 12 for each experimental group) (Supplementary Fig. S1); (ii) sugar consumption during queen rearing in the laboratory (Supplementary Table S1); (iii) fat body content (n = 10, 12, 10, 9 for C, I, N, NI respectively) as an indicator of the nutritional status of queens. The later was used to test if the pesticide induced behavioral changes in workers bees affecting their queens’ attending capacities^29^,^30^,^31^ (Supplementary Fig. S2).

### Queen rearing

**Figure 1:**
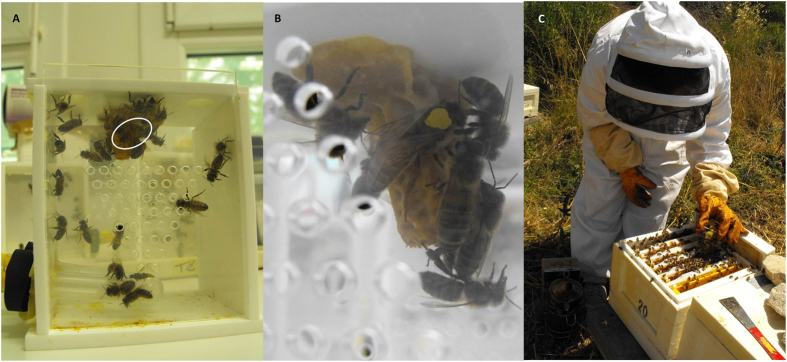
Queen rearing in the laboratory and subsequent transfer to mating nuclei. Experimental cage with an open queen cell stuck on the inside top: queen, attending workers and feeders on the left side bottom are visible (**A**) Detail of a queen color marked over the thorax and a queen cell inside a cage (**B**) Introduction of the queen into the nucleus (**C**).

**Figure 2:**
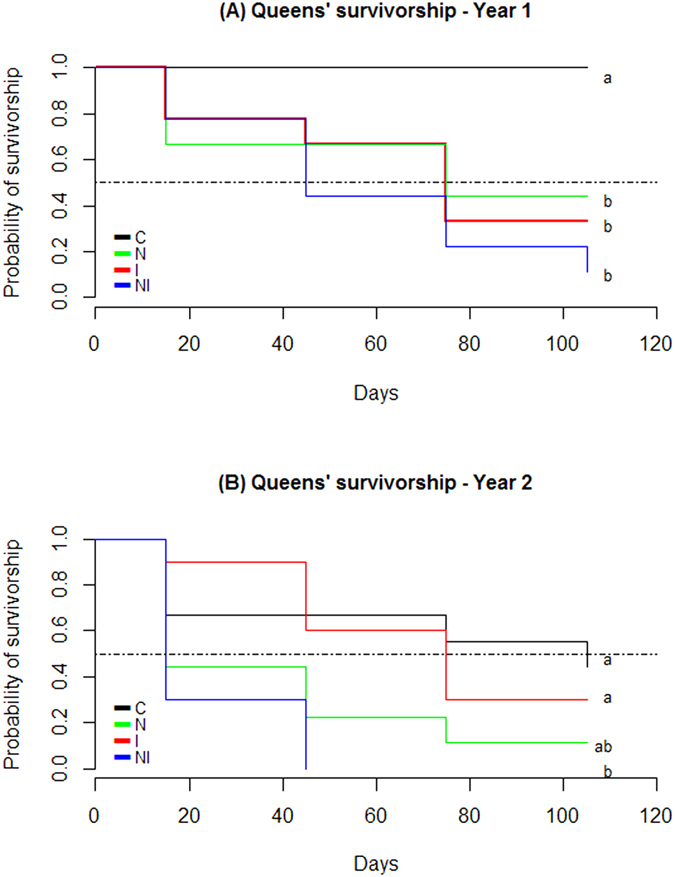
Kaplan Meier survivorship curves of honeybee queens in the field. In laboratory conditions queens were divided in four groups to be parasite-inoculated and pesticide-exposed as follow: *N. ceranae* infected queens (N), imidacloprid exposed queens (I), both infected and exposed to imidacloprid queens (NI) and control queens (C). Queens were then introduced into mating nuclei placed in the field for 3 months. (**A**) Year 1 survivorship curves (n = 8, 9, 9, 9 for C, I, N and NI) and (**B**) Year 2 survivorship curves (n = 9, 10, 9, 10 for C, I, N and NI). Day 0 corresponds to the introduction of queens in the nuclei. Median lifespan (T50) is represented by a dot-dash line. Different letters denotes differences less than 5% of significance. Log-Rank test for 2010: C vs. N: p – value = 0.0151; C vs. I: p – value = 0.0058; C vs. NI: p – value = 0.0002; I vs. N: p – value = 0.774; N vs. NI: p – value = 0.212; I vs. NI: p – value = 0.312; and Log-Rank test for 2012: C vs. N: p – value = 0.0778; C vs. I: p – value = 0.643; C vs. NI: p – value = 0.0046; I vs. N: p – value = 0.0787; N vs. NI: p – value = 0.219; I vs. NI: p – value = 0.001.

**Figure 3:**
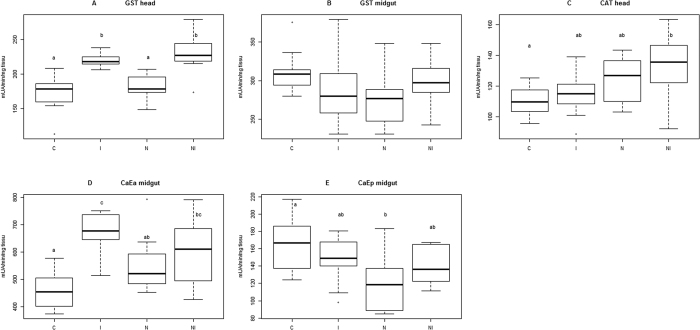
Effect of imidacloprid exposure and *N. ceranae* infection on queens’ tissue enzyme activity. Unmated queens of 8-days-old were analyzed by spectrophotometry to measure GST in the head (**A**) and midgut (**B**) CAT in the head (**C**) CaE *α* (**D**) and CaE *p* (**E**) in the midgut. Four experimental groups were tested: *N. ceranae* infected queens (N), imidacloprid exposed queens (I), both infected and exposed to imidacloprid queens (NI) and control queens (C). Boxplots show 1^st^ and 3^rd^ interquartile range with line denoting median, whiskers encompass 90% of the individuals beyond which outliers are represented by circles. Different letters denote statistically significant differences <5% of significance after ANOVA and Tukey post-hoc test. For C, N, I and NI, n = 11, 12, 11, 9 respectively.

Sister queens were reared according to standard beekeeping methods^32^ by grafting young larvae less than 48-hours-old from a single source colony and reared as queens in a queenless colony^32^. The source colony being different between year 1 and year 2, natural genetic variation has been considered. One to two days before emergence capped queen cells were stuck individually on the inside top of Plexiglas cages (10.5 × 7.5 × 11.5 cm) and kept in incubators at 33 °C. Humidity was maintained between 50–60% through natural evaporation of water in a container. Each queen cell was provided with 30 one-day-old attending workers bees obtained from combs of last stage pupae, which were incubated until emergence and then transferred into the cages (Fig. 1).

### Pesticide exposure

A sublethal dose of the neonicotinoid imidacloprid [1−(6-chloro-3-pyridylmethyl)- N-nitro-imidazolidin-2-ylidene amine] was given to the attending bees and the queen, through contaminated sugar syrup for 10 hours per day. Contaminated sugar syrup was made from a pure standard of imidacloprid (Cluzeau, France) diluted with dimethyl sulfoxide (DMSO) and water to obtain a stock solution of 7 μg/l. The stock solution was aliquoted for daily needs and frozen at −20 °C until use. Syrup was prepared at a concentration of 50% (w/v) sucrose and used to dilute the stock solution to get a final solution of 0.7 μg/l imidacloprid with 0.1% (w/v) DMSO^11^. This concentration was known to cause less than 10% worker bees’ mortality under similar conditions, and no evident effects were observed on physiological markers related to individual and social immunity^11^. The theoretical effective dose of imidacloprid was 0.0083 ng/bee/day for I and 0.0095 ng/bee/day for NI estimated upon the daily consumption of syrup (Supplementary Table S1). Solutions containing sucrose and 0.1% DMSO, were used as controls. The concentration of an aliquot of stock solution was verified from a froze sample by UHPLC-MS/MS. Feeders with sugar syrup were replaced each day at the same time of the day and weighted before and after feeding to estimate syrup consumption. Pesticide exposure began when attending worker bees were introduced in the cages along with the queen cell and continued until queens were 8-days-old, when they were ready to start orientation and mating flights^32^. After daily pesticide exposure, bees were provided *ad libitum* with water, pollen free of *Nosema sp.* spores (checked under microscope), and a sucrose candy (Apifonda^®^ mixed with powder sugar). Food and water were changed every two days.

### *Nosema ceranae* experimental infection of queens

Just after queens emerged in the cages (see queen rearing), they were individually inoculated with spores of *N. ceranae*. For spore inoculation, a solution was prepared containing 50% (w/v) of sucrose in water and a concentration of 100,000 spores/μl in suspension, and each queen bee was fed 2 μl of this solution using a micropipette^18^. From a naturally infected colony, spores of *N. ceranae* were isolated by crushing the abdomens of infected bees in distilled water, filtering the suspension and centrifuging it to collect the spores^33^. Molecular identification of *N. ceranae* in the bees from the infected colony was confirmed by standard PCR^11^. The concentration of spores was estimated with a haemocytometer in the feeding solution and in the midgut of 8-days-old queens to verify the success of infection^32^. Verification was done from 9 up to 12 queens per treatment group from both years (Supplementary Fig. S1).

### Queen introduction in mating nuclei

After *N. ceranae* spore inoculation at birth and exposure to the pesticide in the laboratory for 8 days, each queen was color marked in the thorax according to the experimental group they belong (N, I, NI and C) and introduced into a mating nucleus (Fig. 1). Each year, 40 nuclei (Mini Plus “Stehr”^®^), ten for each experimental group, were established one day before queen’s introduction. To build the nuclei, young workers were collected from the upper hive bodies of large colonies. Upper hives were separated from the brood hive with a wire mesh queen excluder. Each mating nucleus contained approximately 400 g of bees, 4 small frames with foundations, and one feeding frame filled with sugar candy. After introduction in the nuclei queens were left undisturbed for at least 15 days, so they could mate and start lying eggs, which can take place when queens are between 10 to 19-days-old depending on the time of the season^32^. Queens’ acceptance by the bee population from the nucleus was verified fifteen days after introduction. For C groups queens’ acceptance over 65% was considered as satisfactory^19^. Every month, for 3 months, nuclei were carefully observed frame by frame to determine the presence of the queen either by direct observation or by searching for freshly-laid eggs and the presence of brood. We also recorded the unusual presence of drones cells, dead larvae, abnormal brood pattern or workers behavior and signs of diseases or pests. Queen’s survival was the only variable taken into account, since mating nuclei have a reduced space compared to regular hives, therefore less suitable to follow colony development.

The mating hives were set in pairs with their entrances facing opposite directions and painted different colors to facilitate queen’s orientation to return to the correct nucleus.

### Physiological markers

We examined the physiological conditions of 8-days-old queens from the second year, after treatment and just before introduction into the nuclei. For each experimental group, we analyzed the enzymatic activity of CAT in head tissue, GST in midgut and head tissue and CaE *α* and CaE *p* in midgut tissue. Enzyme extraction method was modified from Badiou-Bénéteau *et al*.^23^.

Enzymes were obtained from heads and midguts from queens that were numbed at 4 °C. All tissues were stored at −80 °C until analysis. The analyses were done on each queen individually (for C, N, I and NI, n = 11, 12, 11, 9 respectively). Samples were homogenized at 4 °C with a TissuLyser (Qiagen) (5610 s at 30 Hz) in phosphate buffer pH 7.4 (40 mM sodium phosphate, 10 mM NaCl, 1% (w/v) Triton X-100, containing a mixture of 2 mg/ml of antipain, leupeptin and pepstatin A, 25 units/ml of aprotinin and 0.1 mg/ml of trypsin inhibitor), to make a 10% (w/v) extract. The homogenates were then centrifuged at 15,000 g for 20 min at 4 °C and resulting supernatants were used immediately for analysis of enzyme activities.

GST activity was monitored by following the conjugation of reduced glutathione to 1-chloro-2,4-dinitrobenzene using a method adapted from Habig, Pabst and Jakoby^34^. GST activity was measured by adding enzymatic extract to the reaction mixture containing 1 mM EDTA, 2.5 mM reduced glutathione, 1 mM 1-chloro-2,4-dinitrobenzene and 100 mM Na/K-phosphate pH 7.4. GST activity was followed spectrophotometrically at 340 nm for 5 min at 25 °C.

CAT was measured according to a procedure modified from Beers and Sizer^35^ in a medium containing 30 mM H_2_O_2_ and 100 mM NA-phosphate at pH 7.0. The reaction was monitored by the decrease in absorbance at 240 nm due to the consumption of H_2_O_2_ for 5 min at 25 °C.

CaE *α* (type one) and CaE *p* (type three) are classified according to their substrate specificity corresponding to the hydrolysis of *α* - naphtyl acetate (*α* - NA) and *p* - nitrophenyl acetate (*p* - NA), respectively. For CaE *α* the enzyme reaction was performed for 1 min then stopped with 10% SDS and 4 mg/mL Fast Garnet and kept 10 min in the dark. The reaction product was then measured at 568 nm in a medium containing 0.1 mM *α*-naphtyl acetate as the substrate, 0.01 mM BW284C51 as an AChE inhibitor and 100 mM Na-phosphate pH 7.0. For CaE *p*, the reaction was monitored continuously at 410 nm in a medium containing 0.1 mM *p*-nitrophenyl acetate as the substrate, 0.01 mM BW284C51 as an AChE inhibitor and 100 mM Na-phosphate pH 7.0. Modified by Carvalho *et al*.^22^.

For CaE *α* and *p*, and CAT one unit of enzyme activity was defined as the quantity of enzyme that, under the assay conditions, hydrolysed 1 μmol of substrate per min. For GST, one unit of activity corresponded to the quantity of enzyme conjugating 1 μmol of GSH per min. Results were expressed in terms of tissue activities, as defined above, per quantity of tissue (mUA/min/mg).

### Statistical analysis

Statistical analysis and figures were generated in R version 3.0.2. Survival data was transformed in a Kaplan-Meier table. Log-Rank test was used to compare survival curves using R function {survdiff} and the package {survival}^36^. Survival data from 2012 was not transformed considering the small number of individuals (n = 9 queens in the control group), which brings uncertainty to the normalization of data, keeping the unchanged mortality of controls in the statistical analysis. Enzyme activities were compared between groups with ANOVA and Tukey post-hoc test after assumptions of normality and homogeneity of variance were checked with Shapiro-Wilk test and Bartlett’s test respectively.

## Additional Information

**How to cite this article**: Dussaubat, C. *et al*. Combined neonicotinoid pesticide and parasite stress alter honeybee queens’ physiology and survival. *Sci. Rep.* **6**, 31430; doi: 10.1038/srep31430 (2016).

## Supplementary Material

Supplementary Information
