# Supplementary material for: Combined neonicotinoid pesticide and parasite stress alter honeybee queens’ physiology and survival
Source: Sci Rep. 2016 Aug 31;6:31430. doi: 10.1038/srep31430 (PMC5005999; doi:10.1038/srep31430)
Supplement: Supplementary Information [file srep31430-s1.pdf]

Supplementary Information

Combined neonicotinoid pesticide and parasite stress alter honeybee queens' physiology and survival

Claudia Dussaubat[1,2,4]*, Alban Maisonnasse[1,3,4], Didier Crauser[1,4], Sylvie Tchamitchan[1,4], Marc Bonnet[1,4], Marianne Cousin[1,4], André Kretzschmar[2,4], Jean-Luc Brunet[1,4], Yves Le Conte[1,4]

Affiliations:

[1]INRA, UR 406 Abeilles et environnement, 84914, Avignon, France.

[2] INRA, UR 546 Biostatistique et Processus Spatiaux, 84914, Avignon, France.

[3] ADAPI (Association pour le développement de l'Apiculture), 22, Avenue Henri Pontier, 13326, Aix en Provence Cedex 1, France.

[4] UMT PrADE, Site Agroparc, CS 40509, Avignon, France.

*Correspondence to: cdussaubat@yahoo.com

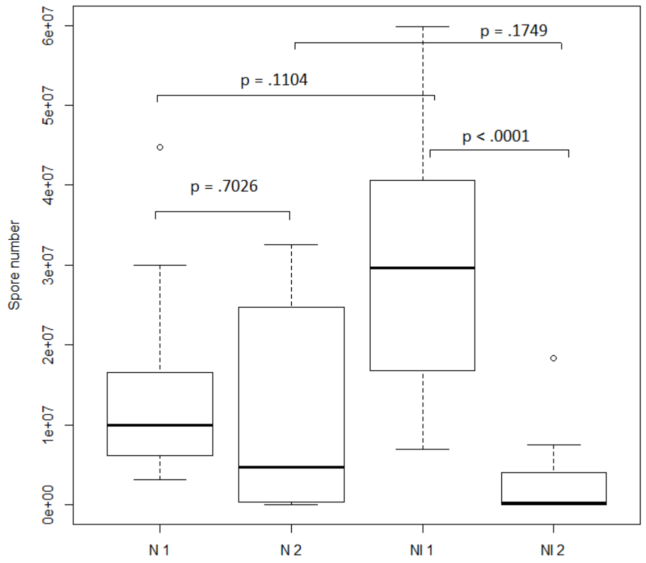

Supplementary Fig. S1. Verification of *Nosema ceranae* infection on queens. Queens were orally inoculated with *N. ceranae* spores at birth and success of infection was verified 8-days post-infection. Infected groups are: N 1 = *N. ceranae* infected queens from year 1, N 2 = *N. ceranae* infected queens from year 2, NI 1 = both queens exposed to imidacloprid and infected with *N. ceranae* from year 1 and NI 2 = both queens exposed to imidacloprid and infected with *N. ceranae* from year 2. Boxplots show 1st and 3rd interquartile range with line denoting median, whiskers encompass 90 % of the individuals beyond which outliers are represented by circles. P- values (p) from Tukey post-hoc test at 95% confidence level after ANOVA are indicated; n = 10, 12, 9, 12 for N1, N2, NI1, NI2 respectively ; assumptions of normality and homogeneity of variance were checked on squared root transformed data with Shapiro-Wilk test and Bartlett's test respectively. Spore levels were similar to [18] under comparable experimental conditions with exception of NI from the second repetition that developed low spore levels compared to the other infected groups but producing a similar effect (Fig. 2). In C no spores were detected with exception of 3 queens from the first repetition, with levels 5 times lower than the dose used to infect experimental queens; in queens from group I no spores were detected.

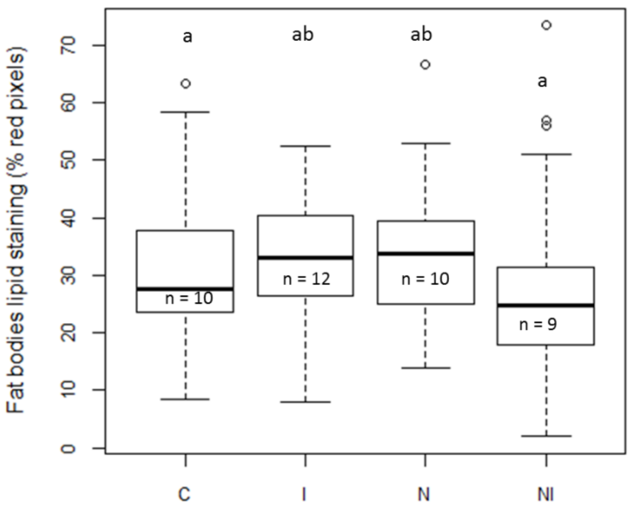

Supplementary Fig. S2. Histological analysis of lipid stores in the fat bodies of 8-days-old queens exposed to imidacloprid and infected with *N. ceranae*. Groups are: I = imidacloprid exposed queens, N = *N. ceranae* infected queens, NI = both queens exposed to imidacloprid and infected with *N. ceranae* and C = control. Boxplots show 1st and 3rd interquartile range with line denoting median, whiskers encompass 90 % of the individuals beyond which outliers are represented by circles. Sample size is indicated in each boxplot. Different letters denote statistical differences < 5% with ANOVA and Tukey post-hoc test at 95% confidence level, assumptions of normality and homogeneity of variance were checked with Shapiro-Wilk test and Levene's test respectively after percentage data was transformed to arsine of squared root (x/100). ANOVA, F = 4.673, p – value = 0.00344; Tukey post-hoc test: C vs. I: p – value = 0.9715, C vs. N: p – value = 0.7868, C vs. NI: p – value = 0.0589, I vs. N: p – value = 0.9482, I vs NI: p – value = 0.0118, N vs NI: p – value = 0.0033. All queens cumulated similar energy reserves presenting then comparable nutritional status after laboratory rearing. Histological analysis of lipid stores in the queens' fat bodies was done using the Oil Red O staining method proved to be efficient in detecting differences in lipid content in honeybee workers and queens [18]. The sternites 3–7 were dissected out of the queens' abdomens where the fat bodies are attached to be then rubbed onto a slide, fixed with 10% formaldehyde, and washed with 60% isopropanol. Then slides were stained with Oil Red O for 15 min and washed with water. The stained tissue was observed under microscope at 400x and 5 pictures of each slide were taken using a CANON Powershot A650 digital camera. Lipid content was quantified by automatically counting red pixels with Adobe Photoshop version 7.0.

Treatment group	Sugar syrup consumption (µg/bee/day)
C	11.54 ± 4.5
I	11.99 ± 4.27
N	12.02 ± 4.38
NI	13.65 ± 4.32

Supplementary Table S1. Consumption of sugar syrup 50% (w/v) per worker bee and per day in micro-grams during queen rearing in cages at the laboratory. The queen and 30 attending workers kept in laboratory cages were fed with sugar syrup contaminated with the pesticide or not during 10 h a day until queens were 8 days old. Syrup consumption was estimated each day based on the difference of feeders' weight before and after the 10 h of exposition. There were 29 cages per treatment from trials carried out on the first year of experiment. Groups are: I = queens exposed to 0.7 µg/l of imidacloprid, N = *N. ceranae* infected queens, NI = both queens exposed to imidacloprid and infected with *N. ceranae* and C = control. Values are mean ± standard error.
